# Task complexity and maximal isometric strength gains through motor learning

**DOI:** 10.14814/phy2.12218

**Published:** 2014-11-26

**Authors:** Jessica McGuire, Lara A. Green, David A. Gabriel

**Affiliations:** 1 Electromyographic Kinesiology Laboratory, Faculty of Applied Health Sciences, Brock University, St. Catharines, Ontario, Canada

**Keywords:** Antagonist coactivation, electromyography, force variability, proprioceptive neuromuscular facilitation, wrist flexion

## Abstract

This study compared the effects of a simple versus complex contraction pattern on the acquisition, retention, and transfer of maximal isometric strength gains and reductions in force variability. A control group (*N* = 12) performed simple isometric contractions of the wrist flexors. An experimental group (*N* = 12) performed complex proprioceptive neuromuscular facilitation (PNF) contractions consisting of maximal isometric wrist extension immediately reversing force direction to wrist flexion within a single trial. Ten contractions were completed on three consecutive days with a retention and transfer test 2‐weeks later. For the retention test, the groups performed their assigned contraction pattern followed by a transfer test that consisted of the other contraction pattern for a cross‐over design. Both groups exhibited comparable increases in strength (20.2%, *P* < 0.01) and reductions in mean torque variability (26.2%, *P* < 0.01), which were retained and transferred. There was a decrease in the coactivation ratio (antagonist/agonist muscle activity) for both groups, which was retained and transferred (35.2%, *P* < 0.01). The experimental group exhibited a linear decrease in variability of the torque‐ and sEMG‐time curves, indicating transfer to the simple contraction pattern (*P* < 0.01). The control group underwent a decrease in variability of the torque‐ and sEMG‐time curves from the first day of training to retention, but participants returned to baseline levels during the transfer condition (*P *< 0.01). However, the difference between torque RMS error versus the variability in torque‐ and sEMG‐time curves suggests the demands of the complex task were transferred, but could not be achieved in a reproducible way.

## Abstract (precis)

This study examines the effect of task complexity on the acquisition, retention, and transfer of increases in maximal strength and decreases in force variability, which is novel. Simple agonist‐only contractions are compared to a more complex reversal contraction pattern as used during proprioceptive neuromuscular facilitation (PNF). The goal was to determine if the more complex contraction pattern interferes with the strength gains and reduced variability by impeding the development of agonist‐antagonist coordination.

## Introduction

To date, there are only two studies that have examined the interaction between task complexity and the acquisition of maximum strength through practice (Gabriel and Kroll 1991; Gabriel et al. 1997). The first study compared agonist‐only contractions to a proprioceptive neuromuscular facilitation (PNF) technique that involved reciprocal contractions, termed the reversal of antagonists. There were only five test sessions, spaced 2 weeks apart, with no other training involved. A control group performed maximal isometric contractions of the elbow flexors while the experimental group performed the following contraction sequence: maximal isometric elbow extension immediately reversing force direction to elbow flexion within a single trial. The study demonstrated that the reciprocal isometric contractions interfered with the expression of maximum strength (Gabriel and Kroll 1991). Using a cross‐over design, the experimental group performed agonist‐only contractions on the last test session to assess transfer, and exhibited an increase in maximum strength comparable to what was acquired by the control group after multiple test sessions (Gabriel and Kroll 1991). The second study showed that the reversal of antagonist did not interfere with strength acquisition but it also did not result in proprioceptive neuromuscular facilitation (Gabriel et al. 1997). Furthermore, both the control and experimental groups exhibited an increase in root‐mean‐square (RMS) amplitude of the agonist and antagonist surface electromyographic (sEMG) activity, but coactivation was not assessed.

Improvement in muscle coordination has been interpreted as a reduction in antagonist coactivation for training regimens consisting of maximal isometric agonist‐only contractions (Carolan and Cafarelli 1992; Geertsen et al. 2008; Laroche et al. 2008; Tillin et al. 2011; Simoneau et al. 2012). It has been speculated that simple planar resistive exercise tasks involving flexion or extension “only” reinforces a reciprocal inhibition pattern that reduces antagonist coactivation with motor learning (Kroll 1981). It is not known if training regimens consisting of the reversal of antagonists contraction pattern can result in a decrease in antagonist coactivation. The complexity of the task may interfere with the reinforcement of a reciprocal inhibition pattern (Gabriel et al. 1997). This is important because motor learning theorists have long predicted decreases in coactivation on the basis that there is a skill component to the expression of maximal strength, where the agonist muscle contracts unimpeded by the antagonist (Kroll 1981).

Furthermore, McGuire et al. (2014) studied maximal isometric contractions across multiple test sessions and showed that alterations in agonist and antagonist muscle activation were used to regulate limb stiffness, which resulted in a decrease in force variability. While the magnitude of agonist muscle sEMG exhibited a progressive increase across test sessions, the magnitude of antagonist sEMG alternated between increases and decreases. The increases and decreases in antagonist sEMG paralleled force variability across test sessions. Force variability was measured two ways: RMS error of maximum force and reproducibility of the entire force‐time curve (variance ratio, VR). The RMS error is analogous to the standard deviation of a window taken from the middle portion of the plateau portion of the force‐time curve which measures task performance. The VR compares the entire force‐time curve for multiple trials on a point‐by‐point basis to assess the reproducibility of motor output. It was hypothesized that the pattern of increases and decreases in antagonist sEMG reflected an iterative process of finding a balance between the two competing functions ascribed to the antagonist: generating sufficient limb stiffness to decrease task variability (Gribble et al. 2003; Osu et al. 2004), while allowing the agonist muscle to contract unimpeded to maximize the expression of force (Kroll 1981).

This article continues the effort to understand the role of motor learning in the expression of maximum strength by studying the effects of task complexity on the acquisition, retention, and transfer of strength gains. Task complexity was manipulated using the reversal of antagonists because it is more complex than agonist‐only contractions and has implications for stroke rehabilitation (Westwater‐Wood et al. 2010; Lu et al. 2011). To this end, a control group performed simple maximal isometric contractions of the wrist flexors (flexion‐only). An experimental group performed complex maximal isometric wrist extension immediately reversing force direction to wrist flexion within a single trial (extension‐to‐flexion). The acquisition phase consisted of three successive test sessions, each spaced 48 h apart. A retention and transfer (crossed‐condition) test occurred during a fourth test session 2‐weeks after the third test session. Wrist flexion torque and sEMG activity of the flexor carpi radials and extensor carpi radialis were monitored concurrently.

## Methods

### Preliminary procedures

Sample size estimation was accomplished using means, standard deviations, and the intraclass reliability coefficient for maximal isometric wrist flexion strength obtained using a measurement schedule similar to that proposed in this study (Kroll 1963). The Case 4 calculations outlined by Cohen (1988) resulted in a sample size of 10 participants per group for a total of 20. However, to protect against the fact that observed error variances may be higher and reliability may be lower, the study recruited 12 participants per group for a total of 24 participants. All participants were college‐aged males. Inclusion criteria were the stated absence of neurological or musculoskeletal disorders of the upper limb, self‐reported right‐hand dominance, and had not performed any forearm resistance training in the past year.

Prior to the first testing session, participants were invited into the laboratory to become familiarized with the nature of the experiment and the equipment. Participants then signed an informed consent document, which outlined the requirements of participation, including the inherent risks, possible benefits, and the right to discontinue at any point in time. All methods and materials were reviewed and approved by the Brock University research ethics board prior to the study (REB#12‐281). Anthropometric data obtained during this preliminary session was used to predict maximal isometric wrist flexion strength. Participants were then ranked and matched on the basis of predicted maximal isometric wrist flexion strength and randomly assigned by pairs into either the control or experimental group. Matching subjects based on predicted maximal isometric wrist flexion strength, rather than after an initial assessment, allowed the first trials to represent initial attempts at the task (McGuire et al. 2014).

### Experimental design

#### Apparatus and testing position

Participants were seated at a testing table at a height that allowed the forearm to rest flat with the elbow at 160° of extension. A custom‐made jig was designed to isolate the hand during isometric contractions of the wrist flexors and extensors. Restraints for the hand were mounted onto a lever arm that was attached to a load cell (JR3 Inc., Woodland, CA). The load cell was secured to the base of the testing table. The back of the upper arm rested on a 20° wedge to maintain the elbow angle. The hand was placed in a half‐supinated position within restraints that contacted the volar and dorsal surfaces. The forearm and hand were placed so that the axis of rotation of the wrist was aligned with the axis of rotation of the lever arm on the load cell. There were restraints for the forearm to minimize extraneous movements. An oscilloscope (VC‐6525, Hitachi, Woodbury, NY) was placed at eye level in front of the participant. The oscilloscope was used to display the torque levels achieved during the contractions.

#### Measurement schedule

The measurement schedule followed has previously been shown to increase maximum isometric strength due solely to motor learning (Kroll 1963). The control group performed maximal isometric contractions of the wrist flexors. The experimental group executed a maximal isometric contraction of the wrist extensors immediately prior to a maximal isometric contraction of the wrist flexors. There were four separate test sessions. The first three sessions were separated by 48 h, and the fourth session occurred 2 weeks after the third session. During the fourth session, participants first performed their assigned contraction pattern to assess retention followed by the crossed‐condition to determine transfer. For the crossed‐condition, the control group performed the more complex contraction pattern (extension‐to‐flexion) while the experimental group completed the simple flexion‐only contractions (Gabriel and Kroll 1991).

To begin each test session, five maximal M‐waves and ten H‐reflexes were evoked in the flexor carpi radialis (FCR). Starting with M‐wave data collection, there were 15‐sec between each evoked potential. Hoffman (H) reflexes were then evoked at 15‐sec intervals, beginning 5‐min after the last M‐wave. The H‐reflexes were only recorded to facilitate location of the V‐wave during strength testing (El Bouse et al. 2013). Another 5‐min rest period preceded maximal isometric strength assessment. Participants then performed 10 trials of their assigned contraction pattern. Each maximal isometric contraction was 5‐sec in duration with 3‐min of rest between each contraction to minimize fatigue (Clarke and Stull 1969). The V‐wave was evoked in the middle of each maximal isometric contraction of the wrist flexors. The protocol for evoking the V‐wave was followed for the three consecutive test sessions and the retention test but not the transfer test.

#### Recording voluntary EMG

At the beginning of each session, the right forearm was prepped for testing. The electrode locations were shaved, cleansed with isopropyl alcohol, and lightly abraded (NuPrep^®^, Weaver and Company, Aurora, CO) to maintain skin‐electrode impedance below 10 kΩ (Grass EZM Electrode Impedance Meter, Astro‐Med Inc., Warwick, RI). Surface electromyographic (sEMG) skin‐electrode impedance was measured before and after the protocol at each test session to ensure it remained below 10 kΩ. The motor points of the flexor carpi radialis (FCR) and extensor carpi radialis (ECR) were then located using a low‐level repeated electrical stimulation on the skin's surface. Once located, these points were marked with indelible ink for electrode placement. Pediatric‐sized electrodes (3 mm electrode diameter, F‐E9M 11 mm, GRASS Technologies, Astro‐Med, Inc.) with an interelectrode distance of 1 cm were placed in a bipolar electrode configuration and used to measure the electrical activity of the FCR and ECR muscles during voluntary and evoked contractions. The electrodes were affixed with two‐sided tape and electrolyte gel (Signa Gel^®^, Parker Laboratories, Fairfield, NJ). A self‐adhesive ground electrode was placed on the dorsal side (back) of the hand.

To ensure the electrode placement was consistent throughout testing sessions, the electrodes were traced with indelible ink. The participants were asked to maintain these tracings between sessions and were welcome to come to the laboratory to have the tracings maintained if needed. Although, maintaining the tracings was helpful for the investigator, it was not necessary. If a participant was unable to maintain a tracing, the location of electrode placement was found using the protocol to locate the motor point that is discussed above. These procedures have been shown to result in high intraclass reliability coefficients suitable for documenting surface electromyographic (sEMG) activity obtained over long periods of time (Calder et al. 2005; Christie et al. 2010).

#### Evoked potentials

The median nerve supplying the FCR was stimulated to obtain the M‐wave, H‐reflex, and V‐wave. Palpating the biceps tendon in the bicipital groove and moving medially located the median nerve; a pulse can be found where the cathode was placed. The cathode and anode were self‐adhesive pad electrodes. The anode was placed on the posterior aspect of the upper arm directly across from the cathode. Both electrodes were connected in series with an isolation unit and a stimulator (Grass Telefactor SIU8 and S88, Astro‐Med Inc., West Warwick, RI), which delivered a square‐wave pulse that was 0.5 ms in duration. The level of stimulation needed to obtain a maximum M‐wave was found by slowly increasing the voltage level until the M‐wave amplitude plateaued and no further increase could be elicited (Tucker and Türker 2007). Supramaximal (110%) stimulation during the voluntary isometric wrist flexion contractions was used to obtain V‐waves, which assessed changes in central drive to the muscle (Aagaard et al. 2002; Aagaard 2003; Vila‐Chã et al. 2012).

#### Instructions to participants

During the voluntary contractions, participants were instructed by the investigator to maximally contract the agonist muscle of each movement. A target line representing the participant's maximum force was presented on the oscilloscope (VC‐6525, Hitachi). This target line served two functions. First, participants were instructed to contract as hard and as fast as possible in order to move their trace to or above the target line. Second, participants were instructed to maintain their force trace parallel to the target line in order to hold a steady force level. Along with the visual feedback presented on the oscilloscope, participants were shown a picture of what an “ideal” force trace looks like to help participants understand the task. Participants were instructed that they were required to use the visual feedback during all maximal voluntary contractions. The visual feedback was only provided during the first three test sessions. The primary method to assess motor learning during retention and transfer is to remove visual feedback. Removing feedback distinguishes between improvements associated with motor skill learning versus performance (Lai and Shea 1999; Kantak and Winstein 2012). The work‐to‐rest ratio for the voluntary contractions was controlled by a tape recording, which cued participants on the timing of contractions. This was provided for all four test sessions. No verbal feedback or encouragement was provided during the testing sessions.

#### Signal processing

All data were collected inside a Faraday cage located in the Electromyographic Kinesiology Laboratory which maintained a signal to noise ratio for sEMG below 20 dB. The sEMG signals were amplified (Grass P511, Astro‐Med, Inc.) to maximize the resolution of the 16‐bit analog‐to‐digital convertor (PCI‐6251, DATAQ Instruments, Akron, OH) and band‐passed filtered (3–1000 Hz). Both force and sEMG signals were digitized at 2048 Hz (DASYLab, DASYTEC National Instruments, Amherst, NH). The force signal was low‐passed filtered (20 Hz, 3 dB) using a fourth order Butterworth digital filter offline in MATLAB (The Mathworks Inc., Natick, MA).

#### Data reduction and criterion measures

**Figure 1.**
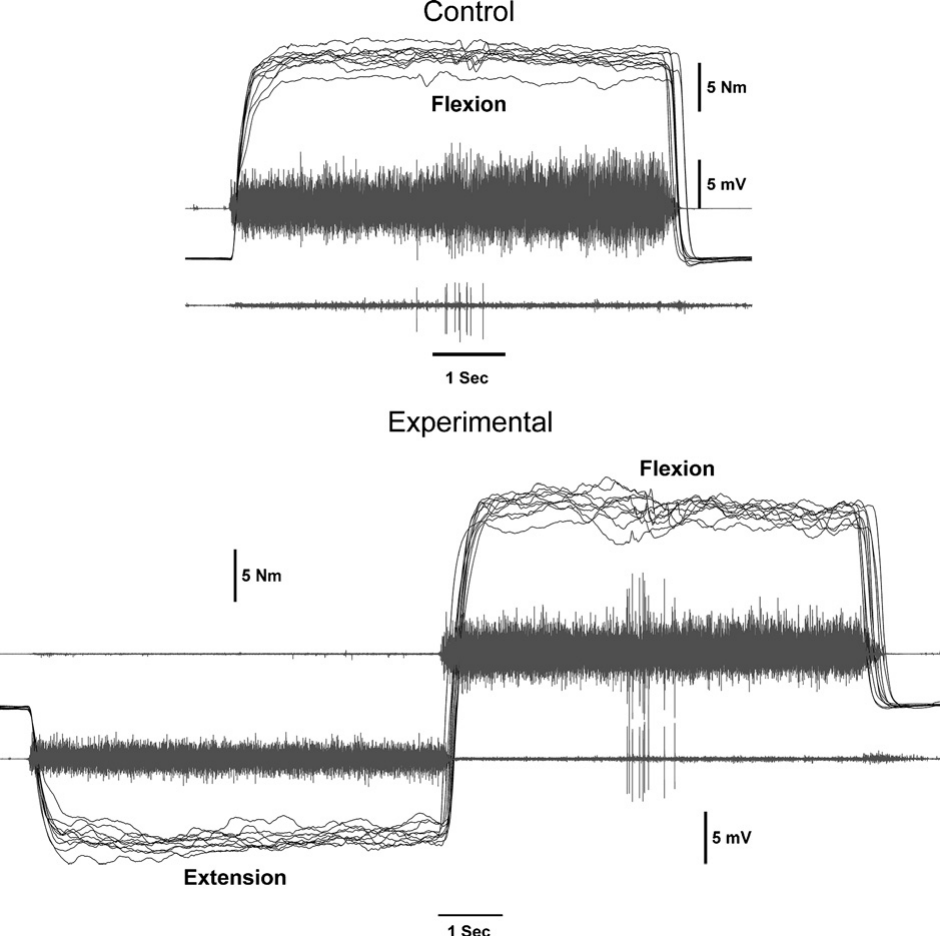
Ten overlapped representative traces for the control (wrist flexion) and experimental (wrist extension‐to‐wrist flexion) groups to illustrate the location of the interpolated twitch to elicit the V‐wave. The 1‐sec window of data was terminated before the evoked potential.

**Figure 2.**
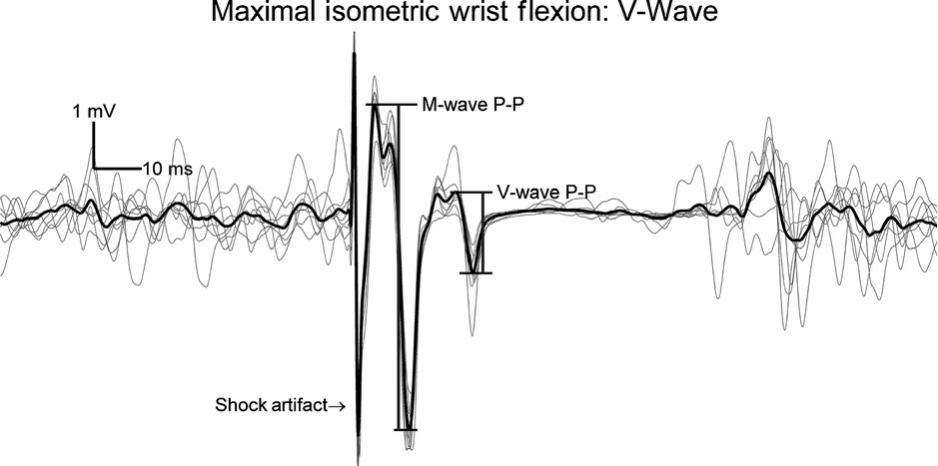
Representative traces to show the peak‐to‐peak (P‐P) amplitudes of the M‐ and V‐waves elicited during a maximal voluntary contraction.

All data reduction was performed using MATLAB software (The Mathworks, Inc.). Figure 1 illustrates raw torque and sEMG traces for both the control (flexion‐only) and experimental (extension‐to‐flexion) conditions. The criterion measures were obtained from a 1‐sec window that terminated immediately before the V‐wave in the middle of each 5‐sec‐wrist flexion contraction. Mean maximal torque, and root‐mean‐square (RMS) sEMG amplitude for the FCR and ECR were calculated from this window. The sEMG measures were used to calculate a ratio for muscle coactivation: ECR antagonist RMS amplitude was divided by FCR agonist RMS amplitude (Kilmer et al. 1982; De Boer et al. 2007). Figure 2 shows the V‐wave P‐P amplitude which was divided by the P‐P amplitude of the maximum M‐wave to calculate V/M_max_ ratio (Aagaard et al. 2002; Del Balso and Cafarelli 2007; Ekblom 2010).

Task variability in maintaining a constant torque was assessed by calculating the RMS error of the middle 3.5 sec of the torque trace. This measure represents the variability of the horizontal portion of the torque trace itself, not relative to the horizontal target line. Prior to the RMS error calculation, the torque trace was normalized to its maximum value. To assess the variability in how the task was generated (motor output), variance ratios (VRs) for torque, and the FCR and ECR sEMG waveforms were calculated. The sEMG VRs for the FCR and ECR waveforms were added to construct a measure of total sEMG variability (Darling et al. 1989; McGuire et al. 2014).

Prior to calculating the VR, the sEMG signals were first rectified then low‐passed filtered at 20 Hz with a zero phase shift fourth order Butterworth digital filter. Torque was similarly filtered at the same low‐pass cutoff frequency. In the case of wrist flexion‐only contractions, the length of each signal started 500 ms before the onset of flexion torque and was terminated 500 ms after torque cessation. The signals were then time‐normalized by interpolating each trace to 8000 data points. The length of the wrist extension‐to‐flexion signals started 500 ms before the onset of extension torque and was terminated 500 ms after flexion torque cessation. Time‐normalization for the wrist extension‐to‐flexion signals involved interpolating each trace to 16,000 data points: 8000 data points for wrist extension and 8000 data points for wrist flexion. The two curves were partitioned based on the inflection point of the first derivative of the torque‐time signal, when the torque curve changed direction from extension to flexion. The VR was then calculated for each block of five trials. The VR was calculated according to the following formula: 
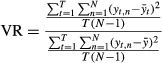


where *T* is the number of data points required (8000), *N* is the number of trials in the VR (five per ratio), *y* represents a single trace with *t* being each point (*t*_1_ is the first point of a single trial), therefore 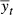 is the average of the five trials at each point, and 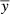 the average mean value.

### Statistical analysis

All statistical procedures were conducted using SAS software (SAS Institute, Cary, NC). Intraclass correlational analysis of variance was conduction on all the measures prior to the main analyses (Christie et al. 2010). Participants completed ten trials each during sessions one through three. During session four, there were five retention trials followed by five trials for the transfer task (cross‐condition) (Damon and Harvey 1987). The first five trials of the consecutive test sessions within the 1 week were designated Blocks 1 through 3, while the retention and transfer tests on session four were designated Blocks 4 and 5 respectively. The transfer test on Block 5 was consistent with a cross‐over experimental design. That is, the control group completed the wrist extension‐to‐flexion contraction pattern while the experimental group performed wrist flexion‐only contractions. A split‐plot (SPF‐*p*.*q*) analysis of variance (ANOVA) with one between‐groups factor (*p *= flexion‐only versus extension‐to‐flexion) and one within‐groups factor (*q *= Blocks) was used to evaluate significant differences. When appropriate, Bonferroni‐corrected orthogonal contrasts were performed for savings analysis to assess retention and transfer. More complex interactions were explored using orthogonal polynomials to evaluate trends in the mean across days (Kirk 2012).

## Results

### Preliminary analyses

#### Participants

The participants’ (*N *= 24) physical characteristics, predicted and observed mean maximal isometric wrist flexion torque, sEMG RMS amplitude for the FCR and ECR for Block 1 are presented in Table 1. Paired samples *t*‐tests revealed no statistical differences between the two groups for any characteristic measure.

**Table 1.** Means (M) and standard deviations (SD) for the physical characteristics of participants in the control and experimental groups

Physical characteristic	Control group (*N*=12)	Experimental group (*N*=12)
	M ± SD	M ± SD
Age (years)	23.42 ± 2.31	23.33 ± 2.31
Height (cm)	179.4 ± 5.89	178.5 ± 5.70
Weight (kg)	79.15 ± 8.65	77.60 ± 8.44
Wrist circumference (cm)	16.76 ± 0.53	16.63 ± 0.88
Predicted peak torque (Nm)	18.59 ± 2.80	17.94 ± 3.36
Torque (Nm) – Day 1	14.40 ± 4.29	12.65 ± 4.74
FCR sEMG (mV) – Day 1	0.31 ± 0.19	0.33 ± 0.22
ECR sEMG (mV) – Day 1	0.14 ± 0.10	0.14 ± 0.05

Significant difference between groups, *P < 0.05.

#### Assessment of cross‐talk

Since coactivity between the FCR and ECR was a main sEMG criterion measure, great care was taken to minimize cross‐talk contamination. One methodological control was to maintain inter‐electrode distance at 1 cm, as recommended by De Luca et al. (2012). A cross‐talk ratio was calculated using the FCR maximum M‐wave as outlined by De Luca and Merletti (1988). The P‐P amplitude of the M‐wave recorded in ECR was divided by the P‐P amplitude of the M‐wave recorded in FCR and the result was multiplied by 100. The percent cross‐talk observed in the ECR was 5.6 ± 2.0% which agrees with the lower value reported by Selvanayagam et al. (2011). The cross‐correlation coefficient was calculated for the sEMG recordings during the voluntary contractions as a follow‐up to the cross‐talk ratio. The amount of common signal (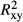) was 2.5 ± 0.5% which is in agreement with Mogk and Keir (2003). Aagaard et al. (2000) considered 2–6% common signal to be sufficiently negligible to allow an evaluation of antagonist coactivation.

#### Statistical assumptions

All statistical assumptions were assessed prior to intraclass correlational analysis of variance to assess reliability of the criterion measures. The RMS error, torque VR, and coactivation ratio measures were skewed and required a log transformation, which was highly effective in restoring a normal distribution to these variables. When Mauchly's test of sphericity was significant, the Greenhouse‐Geisser correction was used (Kirk 2012). The intraclass reliability coefficients for the criterion measures used in this study ranged from *R *= 0.75 to *R *= 0.87, which is considered good to excellent (Merletti et al. 1998; Kollmitzer et al. 1999; Rainoldi et al. 1999).

### Performance of maximal contractions

**Figure 3.**
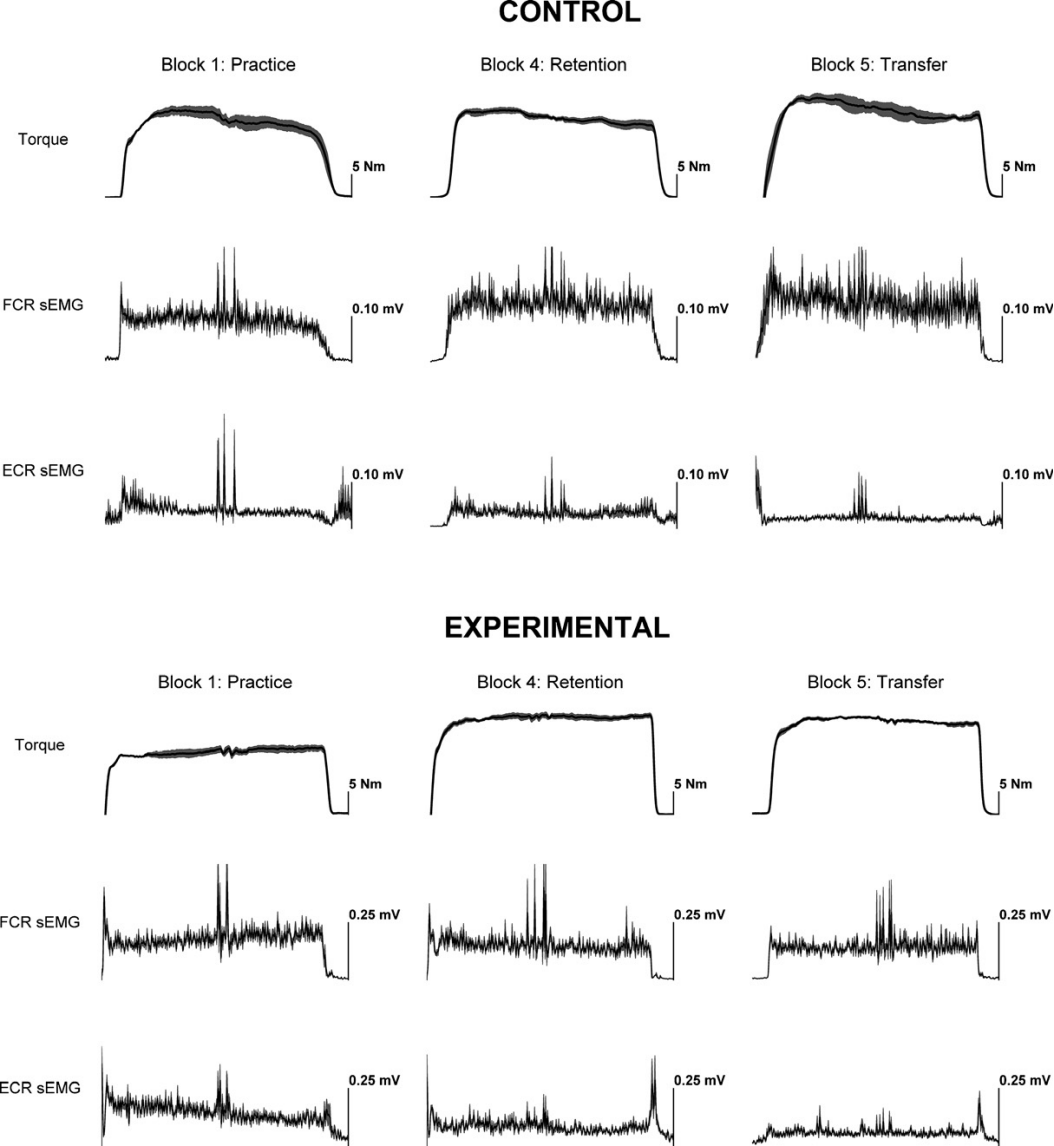
Representative tracings for maximal isometric wrist flexion torque, flexor carpi radialis (FCR) surface electromyographic (sEMG) activity, extensor carpi radialis (ECR) sEMG for the control and experimental groups for Block 1, Block 4 (retention), and Block 5 (transfer). The transfer task was the crossed‐condition for each group: the control group performed the maximal isometric wrist extension‐to‐flexion contraction pattern, while the experimental group completed maximal isometric contractions of the wrist flexors.

Figure 3 shows representative tracings for maximal isometric wrist flexion torque, FCR sEMG, and ECR sEMG for the control (flexion) and experimental (extension‐to‐flexion) groups for Block 1, Block 4 (retention), and Block 5 (transfer) that illustrates essential components of the statistical findings presented in detail below. Both groups exhibited comparable increases in maximal isometric strength and decreases in variability of motor performance (VRs). The same was true for RMS error, not shown. These alterations were retained over the 2‐week interval. The experimental group was able to transfer increases in maximal strength and decreases in variability in torque and sEMG to the simple contraction pattern during the cross‐condition. The control group had more difficulty during the transfer task (extension‐to‐flexion) in terms of torque and sEMG variability, but maximum isometric strength and reduced RMS error were preserved.

The main effect “Between Groups” evaluated overall significant differences between the control group (flexion‐only contractions) versus the experimental group (extension‐to‐flexion contractions). The following grand means and standard deviations are for data across all five blocks. There was no significant (*F*_1,22_ = 3.00, *P *= 0.097) difference in mean maximal isometric wrist flexion torque between the flexion‐only (17.48 ± 4.79 Nm) and extension‐to‐flexion (14.20 ± 5.19 Nm) groups. The main effect Between Groups was not significant (*F*_1,22_ = 0.03, *P* = 0.858) for RMS error. The RMS error for the flexion‐only group was 0.047 ± 0.026 while it was 0.045 ± 0.024 for the extension‐to‐flexion group. Torque VR exhibited no main effect Between Groups (*F*_1,22_ = 0.01, *P* = 0.939). Torque VR was 0.11 ± 0.09 for the flexion‐only group versus 0.11 ± 0.13 for the extension‐to‐flexion group.

The Between Groups main effect was not significant (*F*_1,22_ = 2.12, *P* = 0.159) for total sEMG VR. Total sEMG VR was 1.27 ± 0.29 for the flexion‐only group and 1.41 ± 0.22 for the extension‐to‐flexion group. The coactivation ratio was also not significantly different (*F*_1,22_ = 0.66, *P *= 0.427) between the flexion‐only (0.505 ± 0.375) and extension‐to‐flexion (0.421 ± 0.344) groups. The P‐P amplitude of the V‐wave was extracted only when the waveform could be identified unambiguously. As a result, not all participants had the same number of viable trials across all four test sessions. A complete data set was available for five participants from the flexion‐only group and four participants in the extension‐to‐flexion group. Type IV sum of squares for unbalanced designs was used to evaluate V/M_max_ ratio. There was no significant difference (*F*_1,6_ = 0.03, *P* = 0.870) in V/M_max_ ratio between the flexion‐only (0.27 ± 0.19) and extension‐to‐flexion (0.25 ± 0.15) groups.

It is possible that flexion‐only and extension‐to‐flexion contractions may be comparable with respect the grand mean across blocks. However, the two contraction patterns may have differential effects on retention (Block 4) and transfer (Block 5), which would be evident in statistical testing of the “Group × Block” interaction term. A comparison of the pattern of means across blocks for flexion‐only versus extension‐to‐flexion contractions resulted in a significant (*F*_1,22_ = 2.65, *P* = 0.039) interaction term for torque VR. Orthogonal polynomials were used to analyze differences in trends for the means across blocks between the control (flexion‐only) and experimental (extension‐to‐flexion) groups. The control group exhibited a 51.6% decrease in torque VR between Block 1 and 4. However, the transfer condition (extension‐to‐flexion) caused an increase in torque VR, so that there was only a 1.1% difference between Blocks 1 and 5. The result was a significant quadratic trend that accounted for 98% of variance in means across blocks (*F*_1,11_ = 16.96, *P* = 0.002). In contrast, the experimental group exhibited a 69.3% decrease in torque VR from Block 1 to 5. The linear trend component was significant, which accounted for 75.1% of the variance in means across blocks (*F*_1,11_ = 20.07, *P* < 0.001).

The Group × Block interaction term for total sEMG VR was significant (*F*_1,22_ = 5.00, *P* = 0.001). The difference between the control (flexion‐only) and experimental (extension‐to‐flexion) groups with respect to the pattern of means across blocks mirrored torque VR. The control group exhibited a 12.0% decrease in total sEMG VR between Block 1 and the retention test on Block 4. However, the transfer condition (extension‐to‐flexion) for the control group resulted in an increase in total sEMG VR so that there was only a 0.1% difference between Blocks 1 and 5. The result was a quadratic trend that accounted for 75.3% of variance in means across blocks (*F*_1,11_ = 8.87, *P* = 0.013). In contrast, the experimental group exhibited a slight increase in total sEMG VR of 2.3% between Block 1 and Block 4. Total sEMG VR then decreased 13.8% between Block 4 and Block 5, when the experimental group performed the transfer condition (flexion‐only). The overall pattern of means resulted in linear decrease (*F*_1,11_ = 6.43, *P* = 0.023) that accounted for 33.5% of the variance and a quadratic curvature (*F*_1,11_ = 18.14, *P* = 0.001) that accounted for 41.9% of the variance.

A comparison of the means across blocks for flexion‐only versus extension‐to‐flexion contraction failed to result in a significant Group × Block interaction term for the remaining criterion measures; the *F*‐ratios resulted in *P*‐values ranging from *P *= 0.232 to *P *= 0.870. The following analyses are for the Blocks main effect for scores collapsed across groups (flexion‐only and extension‐to‐flexion). There was a significant (*F*_4,88_ = 9.36, *P* < 0.001) Blocks main effect for mean maximal isometric wrist flexion torque. Orthogonal contrasts for simple effects revealed a significant increase from Block 1 to the retention test on Block 4 (3.81 Nm 20.2%, *P *< 0.001). Savings analysis also revealed that the transfer test on Block 5 was significantly greater than Block 1 (2.41 Nm, 15.1%, *P *= 0.001). There was no significant difference (*P *= 0.509) between Blocks 3 and 4, suggesting the increase in strength was retained over the 2‐week test interval.

The RMS error also exhibited a significant (*F*_4,88_ = 5.74, *P* < 0.001) main effect for Blocks that was explored further with orthogonal contrasts. Savings analysis showed that the RMS error exhibited a 30.5% decrease from Block 1 to the retention test on Block 4 (*P* < 0.001). The reduction in RMS error persisted so that a 26.2% difference was observed between Block 1 and the transfer test on Block 5 (*P *= 0.002). No significant difference was observed between Blocks 3 and 4 (*P* = 0.693), showing that the lower RMS error was retained across the 2‐week test interval.

There was no significant (*F*_3,18_ = 2.44, *P *= 0.098) difference in the V/M_max_ ratio across blocks. In contrast, the coactivation ratio exhibited a significant (*F*_4,88_ = 2.68, *P *= 0.034) main effect for Blocks that was explored further with orthogonal contrasts. Saving analysis revealed a 36.1% decrease from Block 1 to the retention test on Block 4 (*P* = 0.008). The decrease in coactivation ratio was maintained so that there was a 35.2% reduction between Block 1 and the transfer test on Block 5 (*P *= 0.010). No significant difference was observed between Blocks 3 and 4 (*P *= 0.734), suggesting the level of decrease was retained over the 2‐week test interval.

### Muscle coordination

**Figure 4.**
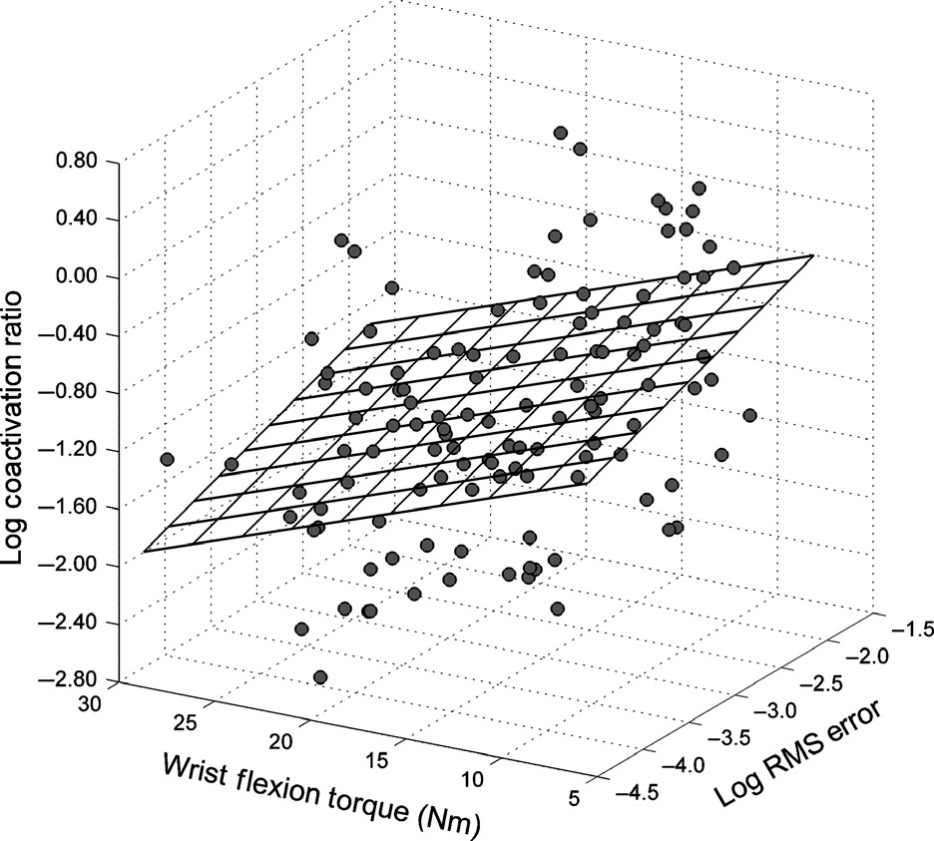
The correlational relationship between maximal isometric wrist flexion torque, the log of the root‐mean‐square (RMS) error of the plateau portion of the torque curve, and the log of the coactivation ratio.

**Figure 5.**
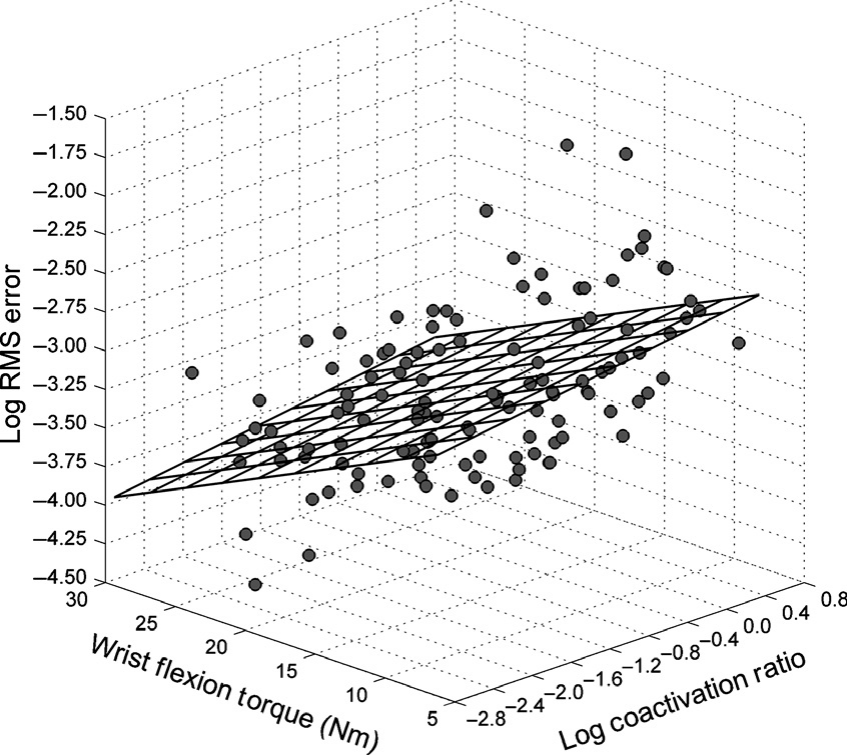
Multiple correlational relationship predicting the log of torque variance ratio (VR) from log of the coactivation ratio and log of total surface electromyographic (sEMG) VR.

Increases in maximal isometric wrist flexion torque were associated with a reduction in both log of RMS error (*r* = −0.51, *P* < 0.001) and the log of torque VR (*r* = −0.47, *P* < 0.001). There was a significant correlation between mean maximal isometric wrist flexion torque and the log of the coactivation ratio (*r* = −0.41, *P* = 0.001). The log of the RMS error had a slightly lower correlation with the log of the coactivation ratio (*r* = 0.34, *P* = 0.001). Figure 4 illustrates the interrelationship between the log of the coactivation ratio, maximal isometric wrist flexion torque, and the log of the RMS error. Decreases in the log of torque VR were associated with decreases in total sEMG VR, so the correlation between the two variables was *r* = 0.44 (*P* < 0.001). The log of torque VR had a slightly lower but significant correlation with the log of the coactivation ratio (*r* = 0.34, *P* < 0.001). Both sEMG variables (coactivation and variance) were used in a multiple regression prediction equation for the log of torque VR. The two sEMG predictor variables resulted in a multiple correlation coefficient of *R* = 0.51 (*R*^2^ = 0.26, *P* < 0.001) which is depicted in Figure 5.

## Discussion

There was no significant difference in the grand means for any the criterion measures obtained between the control (flexion‐only) and experimental (extension‐to‐flexion) groups. The pattern of means across blocks for most of the criterions measures was the same for both groups. The two contraction patterns resulted in an increase mean maximal isometric wrist flexion torque, a decrease in RMS error, a reduction in the coactivation ratio, while the V/M_max_ ratio remained unchanged. These changes were retained over a 2‐week rest interval and transferred during the cross‐over condition. The control (flexion‐only) and experimental (extension‐to‐flexion) groups differed with respect to the torque VR and total sEMG VR. The control group exhibited a decrease in both measures until the retention test, but they returned to initial levels during the cross‐over condition when they performed the more complicated extension‐to‐flexion contraction pattern. The experimental group experienced a progressive decrease in both measures through to the retention test, and the decrease continued during the cross‐over condition when they performed the more simple flexion‐only contractions. The following paragraphs will discuss the theoretical implications of the results.

Consistent with the earlier findings of Gabriel et al. (1997), the extension‐to‐flexion contraction pattern did not interfere with the learning‐related increases in maximum isometric strength. In the present study, when averaged across both groups, there was a 20.2% increase in maximal isometric wrist flexion strength. The increase in maximum isometric wrist flexion strength was retained over a 2‐week rest period and transferred to the new task (cross‐over condition). Our findings also corroborate the observations of McGuire et al. (2014). Increases in strength were accompanied by a decrease in RMS error. The decrease in RMS error was evident during both the retention test and transfer to the new task (cross‐over condition). The relative permanence of increased strength and decreased RMS error, and the transfer of increased strength and decreased RMS error to the new task (cross‐over condition), is evidence that motor‐skill learning had occurred (Kohl and Guadagnoli 1996; Etnier and Landers 1998; Lai and Shea 1999; Wright and Shea 2001; Kantak and Winstein 2012). Further support for motor skill learning is given by the fact that any gains associated with physiological adaptations due to a limited number of contractions would have dissipated over the 2‐week interval (Häkkinen and Komi 1983; Mujika and Padilla 2001).

The fact that the control group transferred the reduced RMS error to the more complex contraction pattern is consistent with the work of Wulf and Shea (2002). The authors suggested that transfer from a simple to more complex task would be facilitated if the demands of the simple task are high (Wulf and Shea 2002). Participants had to attend to an aural stimulus from a tape recording that provided cues for the timing of contractions throughout all phases of the trial. At the same time, they had to monitor an oscilloscope to obtain feedback about task performance in terms of maintaining the plateau portion of the torque trace horizontal to a target line. All of these factors combined to heighten arousal, which enhanced information processing related to task learning (Dimitrijevic et al. 1992; Lai and Shea 1999; Guadagnoli and Kohl 2001; Sherwood and Lee 2003).

The V‐wave was measured to assess changes in neural drive to the FCR. Contrary with the observations of Aagaard et al. (2002) and Vila‐Chã et al. (2012), we observed no significant differences in the V/M_max_ ratio between groups or across blocks. Participants in the present work performed far fewer contractions (i.e., 30) prior to the rentention test, compared to short‐term resistance exercise studies that required several hundred contractions (Carolan and Cafarelli 1992; Aagaard et al. 2002; Kamen and Knight 2004). It is possible that the lack of change in V/M_max_ ratio reflects a dose‐response effect where 30 maximal effort contractions were insufficient for significant changes in neural drive to the FCR.

The present work demonstrated a reduction in the coactivation ratio across blocks for both groups. A reduction in the coactivation means that decreases in the relative activation of the ECR reduced antagonist muscle opposition, leading to an overall increase in net joint torque (Buchanan et al. 1993; Ramsay et al. 2009). The lack of change in the V/M_max_ ratio of the agonist, in addition to, a significant reduction in the coactivation ratio, suggests that the increase in maximum strength was primarily due to a reduction in ECR opposition to FCR force output. These findings support the hypothesis of Patten et al. (2001) who suggested that the earliest adaptions to resistive exercise task are associated with skill learning and involve agonist‐antagonist coordination. The idea that the reduction in the coactivation ratio led to an increase maximum wrist flexion torque is supported by the significant negative correlation (*r* = −0.41) between the two variables.

While the present results support the role of motor learning in resistive exercise, the more complex contraction pattern (extension‐to‐flexion) did not infer with the development of muscle coordination as predicted (Kroll 1981). We believe that the experimental group exhibited adaptations comparable to the control group, because they were given a sufficient number of contractions within each session to develop an internal model for successful task completion, which was refined and updated across the three consecutive sessions (McGuire et al. 2014). Using the same experimental set‐up for maximal isometric elbow flexion, McGuire et al. (2014) showed that, when participants were given a large number of contractions (massed) within a test session, they were better able to develop an internal model of the task.

The participants in the study by McGuire et al. (2014) increased maximal isometric strength while decreasing RMS error. In that study, the correlation between RMS error and total sEMG activity of the elbow muscles (biceps + triceps) was *r* = −0.94. While the variability of the biceps sEMG waveform exhibited a progressive decrease with increases in maximum strength, variability of the triceps sEMG waveform alternated between increases and decreases along with changes in RMS error. It was theorized that these changes in triceps sEMG activity reflected an iterative process of finding a balance between two competing functions ascribed to the antagonist: generate sufficient limb stiffness to decrease RMS error (Gribble et al. 2003; Osu et al. 2004), while allowing the agonist muscle to contract unimpeded to maximize the expression of force (Kroll 1981). The correlation results support the hypothesis that muscle coordination involves achieving “minimally sufficient” antagonist coactivation that would serve both functions. The negative correlation (*r* = −0.41) between maximal isometric torque and the coactivation ratio in addition to the positive correlation (*r* = 0.34) between RMS error and the coactivation ratio suggests that participants learned to achieve this balance. Torque VR predicted from both the coactivation (*r* = 0.34) ratio and total sEMG VR (*r* = 0.44) accounted for 26% of the variance, further suggests that regulating the balance was important for motor output variability.

McGuire et al. (2014) previously demonstrated that RMS error and torque VR can exhibit different patterns of change in response to the same measurement schedule for maximal isometric contractions. The same phenomenon was once again observed in the present investigation. The RMS error exhibited a progressive decrease regardless of complexity of the contraction pattern, which was retained and transferred during the crossed‐condition. In contrast, the control group exhibited a marked increase in torque and sEMG VRs during the crossed condition. Guadagnoli et al. (1996) stated: “Theorists have suggested that participants’ primary concern early in practice is to understand what it to be done and how performance is evaluated, rather than determining the most efficient way of meeting the task demands.” The continued decrease in RMS error for the control group simply reflects a transfer of understanding the demands of the task while the higher VR merely reflects the beginning of an iterative process associated with a new contraction pattern (Proteau et al. 1992; McGuire et al. 2014). Müller and Sternad (2004) suggest that it is possible for subcomponents of a task to exhibit different levels of variability that can improve at different rates.

## Implications

It has been demonstrated the reversal of antagonist technique does not interfere with motor learning‐related increases in maximum isometric contraction if a sufficient number of contractions are administered within each practice session. The next step is to determine if PNF is effective when there is a deficit in muscle activation as might exist in an older adult population (Kroll 1972; Kamen et al. 1995; Connelly et al. 1999; Patten and Kamen 2000). Since task complexity of isometric contractions can reduce motor output in older adults (Barry et al. 2005), it would be important to determine if a reversal of antagonists contraction pattern would be sufficient to produce comparable strength gains to agonist‐only contractions. Using isometric contractions, Onushko et al. (2014) also showed that practicing with easier tasks might be advantageous to improve motor learning in older adults. This is particularly relevant because Chen et al. (2014) recently demonstrated that older adults had impaired motor learning‐related alterations in antagonist coactivity.

## Conflict of Interest

None declared.
